# Risk Indicators for Urinary Tract Infections in Low Risk Pregnancy and the Subsequent Risk of Preterm Birth

**DOI:** 10.3390/antibiotics10091055

**Published:** 2021-08-31

**Authors:** Dominique E. Werter, Brenda M. Kazemier, Caroline Schneeberger, Ben W. J. Mol, Christianne J. M. de Groot, Suzanne E. Geerlings, Eva Pajkrt

**Affiliations:** 1 Department of Obstetrics and Gynecology, Amsterdam UMC, University of Amsterdam, Meibergdreef 9, 1105 AZ Amsterdam, The Netherlands; b.m.kazemier@amsterdamumc.nl (B.M.K.); cj.degroot@amsterdamumc.nl (C.J.M.d.G.); e.pajkrt@amsterdamumc.nl (E.P.); 2 Department of Microbiology, Amsterdam UMC, University of Amsterdam, Meibergdreef 9, 1105 AZ Amsterdam, The Netherlands; c.schneeberger@amsterdamumc.nl; 3 Department of Obstetrics and Gynecology, Monash University, 27 Rainforest Walk, Melbourne, VIC 3800, Australia; b.w.mol@amsterdamumc.nl; 4 Department of Internal Medicine: Infectious Diseases, Amsterdam UMC, University of Amsterdam, Meibergdreef 9, 1105 AZ Amsterdam, The Netherlands; s.e.geerlings@amsterdamumc.nl

**Keywords:** asymptomatic bacteriuria, preterm birth, recurrent urinary tract infections, risk indicators, urinary tract infections

## Abstract

Symptomatic urinary tract infections are associated with preterm birth. However, data on risk indicators for urinary tract infections are limited and outdated. The research is a secondary analysis. The study was a prospective multicenter cohort study of low-risk pregnant women. Logistic regression was used to identify risk indicators for urinary tract infections. The incidence of urinary tract infections was 9.4%. Multivariate logistic regression showed that a history of recurrent urinary tract infections and the presence of asymptomatic bacteriuria in the present pregnancy were associated with urinary tract infections (resp. OR 3.14, 95%CI 1.40–7.02 and OR 1.96 95%CI 1.27–3.03). Women with a urinary tract infection were at increased risk of preterm birth compared to women without a urinary tract infection (12 vs. 5.1%; adjusted HR 2.5 95%CI 1.8–3.5). This increased risk was not found in women with the identified risk indicators (resp. 5.3% vs. 5.1%, adjusted HR 0.35 95%CI 0.00–420 and adjusted HR 1.5 95CI% 0.59–3.9). In conclusion, in low-risk pregnant women, risk indicators for urinary tract infections are: a history of recurrent urinary tract infections and the presence of asymptomatic bacteriuria. The risk of preterm birth is increased in women with a urinary tract infection in this pregnancy. However, women with recurrent urinary tract infections and asymptomatic bacteriuria this pregnancy appear not to be at increased risk of preterm birth.

## 1. Introduction

Preterm birth is the leading cause of perinatal mortality and morbidity [1,2]. Preterm birth is directly responsible for the death of around one million neonates worldwide every year and is known to cause long-term neurologic and developmental disabilities [2,3,4]. Symptomatic urinary tract infection (UTI) in pregnancy is associated with preterm birth [5,6,7]. UTI is defined as the presence of bacteriuria with symptoms such as dysuria, frequent urination or lower abdominal pain. Previous research has shown that the incidence of UTI during pregnancy varies between 2.3% and 15% [6,8,9]. Since prevention of UTI may be helpful in preventing preterm birth, identification of women who are more prone to develop a UTI is required.

Unfortunately, epidemiological data on risk indicators for UTI during pregnancy are limited or outdated. Current knowledge is largely based on studies performed in the sixties, seventies and eighties [10,11,12]. More recently published research was predominantly performed in non-western countries [13,14,15,16]. Consequently, there is a paucity of data on current risk indicators in modern western obstetric populations.

The aim of this study is to identify potential risk indicators for the development of a UTI in pregnancy. In addition, we explored whether the preterm birth risk was increased in women with these risk indicators.

## 2. Results

Between October 2011 and August 2013, a total of 5621 women were included in the ASB trial. In the ASB trial presence of bacteria were tested using a dipslide (an alternative for ordinary culture consisting of a plate with two culture media: cysteine lactose electrolyte deficient medium and MacConkey. With a sensitivity of 98%, and a specificity of 99.6%). ASB in this trial was defined as ‘the isolation of bacteria in at least 1 × 10^5^ colony-forming units per mL of cultures urine in the absence of signs or symptoms of a UTI [17]. Of these women, 489 women were excluded from the trial for several reasons first of all because of symptoms of a UTI at the time of screening (*n* = 163), secondly because a dipslide [17] was not performed (*n* = 113), furthermore because of an increased risk for complicated UTI or preterm birth (*n* = 110) or because of antibiotic use within two weeks of screening (*n* = 103) [17]. Another 214 women were lost to follow up. Finally, 4918 women were analyzed.

Baseline characteristics of the cohort are presented in Table 1.

The main outcome was risk indicators of UTI treated with oral antibiotics. Among 4918 women, 463 (9.4%) women developed a UTI. The univariate logistic regression analysis for UTI identified seven statistically significant risk indicators for a UTI; maternal age, non-Caucasian ethnicity, not living with a partner, low education, smoking, having a history of recurrent UTI and presence of asymptomatic bacteriuria (ASB) around 20 weeks of gestation (Table 2).

In the multivariate logistic regression only two risk indicators remained statistically significant associated with UTI. Having a history of recurrent UTI tripled the risk of the development of a UTI odds ratio (OR) 3.14, 95% confidence interval (95%CI 1.40–7.02) and having ASB this pregnancy around 20 weeks almost doubled the risk of a UTI during pregnancy (OR 1.96 95%CI 1.27–3.03) (Table 3).

Of all women with UTI, 73 women (16%) had one or both risk indicators. Of the women with UTI 19 (4.1%) had a history of recurrent UTI and 57 (12%) had ASB. Of the women without UTI, 56 (1.3%) had a history of recurrent UTI and 261 (5.9%) had ASB.

Table 4 shows the risks of preterm birth and the corresponding adjusted hazard ratio (HR) for preterm birth in women with UTI this pregnancy and separate for women with and without the risk indicators for UTI. Women with a UTI this pregnancy had an increased risk for preterm birth (12% preterm births, adjusted HR 2.5, 95%CI 1.8–3.5). In a subgroup of women with a history of previous UTI and a UTI in the present pregnancy, no increased risk of preterm birth was found (5.3% preterm births, adjusted HR 0.35 95%CI 0.00–240, Table 4). In the subgroup of women with ASB and a UTI in the present pregnancy, no significantly increased risk of preterm birth was found either (9% preterm births, adjusted HR 1.5 95%CI 0.59–3.9, Table 4).

## 3. Discussion

We found that risk indicators independently associated with UTI during pregnancy were a history of recurrent UTI and the presence of ASB. The risk of preterm birth was increased in women with a UTI during the current pregnancy. However, this increased risk appeared not to be existing in a subset of women with a history of recurrent UTI or the presence of ASB in the present pregnancy and a UTI in the present pregnancy.

A history of UTI is found to be associated with an increased risk for UTI outside of pregnancy [18,19]. Several other studies showed that a history of UTI is also associated with UTI amongst pregnant women [20,21,22]. However, most of these studies were performed in developing countries or a long time ago.

In our study having ASB was found to be a risk indicator for the development of a UTI, also after adjustment, which is in line with previous studies showing that having ASB increases the risk of new episodes of ASB, the development of UTI or pyelonephritis [17,19]. In several countries around the world, screening for ASB is still recommended. The reason for this screening is the possible association between ASB and pyelonephritis as well as ASB and preterm birth [23].

In our previous publication we found no association between preterm birth and ASB [17]. However, an association between UTI and preterm birth was found in this present study. This could be explained by the fact that only a small proportion of women with UTI in this pregnancy had ASB. In addition, not all women with ASB developed a UTI. This explains why UTI can be associated with preterm birth, whereas ASB in itself was found not to be associated with preterm birth [17].

We found a trend towards a lower preterm birth rate in women with a UTI during the present pregnancy and a history of recurrent UTI. Previous studies have shown that bacterial and host factors could be different in women with recurrent UTI compared to women with non-recurrent UTI. In non-pregnant women it was shown that the pathway leading to UTI is different in women who have recurrent UTI. It could be hypothesized that the immunological reaction of the (pregnant) body against UTI is less strong when it already experienced a UTI before. It could be that uropathogens causing a first UTI are more virulent than uropathogens causing a recurrent UTI [24,25]. On the other hand, certain polymorphisms in the host are associated with an increased risk of bacteriuria due to deficiencies in the recognition of pathogens which result in an inadequate immune response [26,27]. An explanation for the found results could be that the inadequate immune response as result of the abovementioned mechanisms, in women with recurrent UTI, could result in less prostaglandin release and subsequently less preterm birth. Future studies should investigate whether there is a difference in prostaglandin release in women with a first versus a recurrent UTI.

An alternative methodological explanation can be that women with recurrent UTI are treated pragmatically with antibiotics for their UTI, simply because they developed symptoms in accordance with UTI and without prior confirmation of the diagnosis. Perhaps not all the women with recurrent UTI had a culture proven UTI and these women are misclassified. Pregnancy can mimic the complaints of a UTI, like frequency. This could be one of the reasons why there is no association found in women with recurrent UTI.

The strengths of this study are the design with prospectively collected data and the relatively large sample size in a country where screening and treatment of ASB is not recommended [28].

Baseline characteristics, exclusion criteria and possible risk indicators used in this study were self-reported by the women before they handed in the dipslide. Recall bias may have occurred for instance for the history of previous UTI. Although women were able to fill out the questionnaire in privacy, the prevalence of smoking, alcohol use and drugs use during pregnancy could be underestimated since women may tend to “please” the caregiver with their answer. Since not all caregivers ask about current use of antibiotics or symptoms of a UTI at the time of screening, we decided to add these items to the questionnaire. Women’s own knowledge about UTI and treatment have shown to be limited [29]. Moreover, it is also possible that women confused antifungal treatment for antibiotic treatment leading to unjust exclusion for the study. Because of the before mentioned reasons both over- and underestimation of UTIs could occur. We do not think that the embedded RCT, in the original trial, has too much influence on our data, since the percentage of women receiving antibiotics for ASB the RCT is limited (*n* = 40). Unfortunately we were not able to collect the cultures and susceptibility patterns of the bacteria, since most of the urine cultures were collected at the general practitioners and not in the hospital. We therefore miss the susceptibility patterns of the pathogens, with the current rise of antibiotics resistance this information becomes more and more important.

## 4. Materials and Methods

### 4.1. Study Design

This study is a secondary analysis of the ASB study. The ASB study was a national multicenter prospective cohort study with an embedded double-blind placebo-controlled trial. The study included pregnant women from eight hospitals and five ultrasound centers across the Netherlands, after they gave written informed consent [17]. The representability of the sample was increased by the fact that the different hospital and ultrasound centers were geographically spread, with a population with a large diversity in socioeconomic status and different levels of obstetric care. Women with a history of preterm birth before 34 weeks gestational age, warning signs of an imminent preterm birth, major fetal congenital malformations, antibiotic use within two weeks of screening, known G6PD deficiency, hypersensitivity to nitrofurantoin, or risk indicators for complicated UTI were excluded (such as pre-gestational diabetes mellitus or functional or structural abnormalities of the urinary tract) [17].

Women, 18 years old or older, with a singleton pregnancy, without symptoms of a UTI were offered the possibility to be screened ASB between 16 and 22 weeks of gestation. A dipslide was used to identify ASB [17]. Currently in the Netherlands, pregnant women are not routinely screened and treated for ASB during pregnancy [28].

All participating women filled out a questionnaire containing questions about baseline characteristics, risk factors and exclusion criteria [17].

The study was stopped prematurely when 4918 women were included, after the planned interim analysis. This was due to the low incidence of the primary outcome [17]. More detailed information on methods and outcomes has been previously described [17]. For the purpose of this analysis, we divided the cohort into two groups; women with and without a UTI during pregnancy.

### 4.2. Definitions and Demographic Variables

The main outcome was risk indicators for UTI during pregnancy. UTI was defined as a clinical report of the participant herself of symptoms such as dysuria, frequent urination or lower abdominal pain, treated with oral antibiotics. Since the majority of the UTIs in pregnant women in the Netherlands are diagnosed and treated by the general practitioner instead of the obstetrician, it was not possible to obtain microbiological confirmation of bacteriuria in all cases. Furthermore the susceptibility patterns of the bacteria were not collected.

Univariable logistic regression was used to identify possible risk indicators associated with the development of UTI. Risk indicators investigated were maternal age, non-European ethnicity, primigravida, short inter-pregnancy interval (<1 year), not living with a partner, body mass index (BMI), low education, smoking, alcohol use, drugs use, history of recurrent UTI, comorbidity (thyroid disease, lung disease, psychiatric disease, neurologic disease, cardiovascular diseases and (pre-existing) high blood pressure), conception with assisted reproductive technology and ASB. A history of recurrent UTI was defined as a history of three or more previous UTIs before this pregnancy.

The secondary outcome was the risk of preterm birth in women with and without significant risk indicators for UTI. Preterm birth was defined as a delivery before 37 weeks of gestational age.

### 4.3. Statistical Analysis

In case of missing data, multiple imputations were used to generate possible values for missing data, thereby creating ten “complete” sets of data [30]. Both patient characteristics and outcomes were taken into account to impute missing data. We used an iterative Markov chain Monte Carlo method for the generation of missing values and created ten imputed datasets and the pooled estimates.

In the multivariable logistic regression analysis, all variables that were statistically significant (*p* ≥ 0.05) were retained in the model to evaluate conditional associations between the characteristics and UTI. The associations were expressed as adjusted OR with 95%CI.

Adjusted HR were created to investigate the association of UTI and preterm birth, both with and without the found risk indicators for UTI. All analyses were performed using IBM SPSS Statistics, version 25 (IBM, Chicago, IL, USA).

### 4.4. Ethical Approval

The ASB study was conducted in accordance with the Declaration of Helsinki. The ASB study was approved by the research ethics committee of the Academic Medical Centre, Amsterdam, the Netherlands (approval number MEC 2011-073, date of approval 29-4-2011) and by the institutional review board of each participating hospital. The national perinatal registry in the Netherlands (PERINED) approved linkage of the ASB cohort with their database to further complete missing data on outcomes (approval number 13.64, date of approval 17-12-2013).

### 4.5. Trial Registration Number and URL of the Registration Site

Dutch Trial Registry, number NTR3068 (https://www.trialregister.nl/trial/2921 accessed on 7 March 2021). The date of initial participant enrollment was 11 October 2011.

## 5. Conclusions

Two independent risk indicators were identified for the development of a UTI namely having a history of recurrent UTI and having ASB around 20 weeks of gestational age. However, only a small number of women with a UTI also had one of the identified risk indicators. The risk of preterm birth was increased in women with a UTI during pregnancy, however this risk appeared not to be existing in a subset of women with a history of recurrent UTI too and with ASB in the present pregnancy. Future research should focus on the consequences of UTI and evaluate whether women with recurrent UTI are more protected against the increased risk of preterm birth, or if this is only a result of misdiagnosed recurrent UTIs due to mimicking of the symptoms. Next to that, the role of different pathogens should be further investigated. The impact of one pathogen on pregnancy can be different than the impact of another uropathogen. In future research it is of high importance to take the type of pathogen and its pathogenicity into account.

## Figures and Tables

**Table 1:** Baseline characteristics.

	*n* = 4918	%
Maternal age, mean (SD)	31.2	
Caucasian ethnicity	4086	83
Primigravida	1990	40
Not living with a partner	411	8.4
Pre-pregnancy BMI, median (IQR)	23.6	
Morbid obesity (BMI ≥ 30)	385	7.3
Low education	492	10
Smoking	318	6.5
Alcohol use	72	1.5
Drugs use	11	0.22
More than 3 previous UTI	75	1.5
UTI this pregnancy	463	9.4
Comorbidity Thyroid disease	113	2.3
Lung disease	116	2.4
Psychiatric condition	97	2.0
Neurologic disorder	91	1.9
Cardiovascular disease	28	0.57
High blood pressure (pre-existing)	50	1.0
Conception after IVF/ICSI ^$^	135	2.8

BMI: Body mass index; ^$^ IVF/ICSI: in vitro fertilization/intracytoplasmic sperm injection.

**Table 2:** Univariate logistic regression to identify possible risk indicators of UTI.

	UTI	%	NO UTI	%	OR	95%CI	*p*-Value
	*n* = 463	9.4	*n* = 4455	90.6			
Maternal age, mean (SD)	30.2		31.3		0.95	0.93–0.97	0.01
Caucasian ethnicity	367	79	3719	83	0.75	0.58–0.99	0.02
Primigravida	205	44	1784	40	1.19	0.97–1.46	0.08
If multiparous; inter pregnancy interval ≤ 12 months	65	25	635	24	1.08	0.78–1.50	0.90
Not living with a partner	58	13	354	8.0	1.64	1.14–2.36	0.00
Pre-pregnancy BMI, median (IQR)	23.6		23.7		1.00	0.97–1.02	0.70
Morbid obesity (BMI ≥ 30)	30	6.5	354	8.0	0.81	0.54–1.21	0.26
Low education	56	12	337	7.6	1.67	1.21–2.31	0.00
Smoking	50	11	267	6.0	1.90	1.35–2.68	0.00
Alcohol use	7	1.5	64	1.4	1.06	0.44–2.58	0.90
Drugs use	1	0.22	10	0.22	1.00	0.13–7.94	0.97
More than 3 previous UTI	19	4.1	56	1.3	3.37	1.87–6.08	0.00
Comorbidity Thyroid disease	15	3.2	98	2.2	1.50	0.84–2.66	0.16
Lung disease	16	3.5	100	2.2	1.56	0.89–2.71	0.10
Psychiatric condition	9	1.9	88	2.0	0.95	0.40–2.22	0.96
Neurologic disorder	12	2.6	79	1.8	1.43	0.76–2.68	0.21
Cardiovascular disease	2	0.43	26	0.58	0.74	0.17–3.14	0.68
High blood pressure (pre-existing)	6	1.3	44	0.99	1.41	0.60–3.30	0.53
Conception after IVF/ICSI ^$^	12	2.6	123	2.8	0.94	0.49–1.80	0.83
ASB	57	12	261	5.9	2.25	1.65–3.06	0.00

BMI: Body mass index; ^$^ IVF = in vitro fertilization, ICSI = intracytoplasmic sperm injection.

**Table 3:** Multivariate logistic regression to identify risk indicators of UTI.

	OR	95%CI
Maternal age, mean (SD)	0.98	0.95–1.01
Caucasian ethnicity	0.87	0.59–1.28
Not living with a partner	1.10	0.64–1.91
Low education	1.20	0.72–1.99
Smoking	1.50	0.93–2.44
More than 3 previous UTI	3.14	1.40–7.02
ASB	1.96	1.27–3.03

**Table 4:** Risk of preterm birth in women with UTI.

	N	Preterm Birth < 37 Weeks N (%)	Adjusted HR	95%CI
No UTI	4455	229 (5.1)	Reference	
All UTI	463	56 (12)	2.5	1.8–3.5
UTI without ASB or recurrent UTI	390	50 (13)	2.6	1.9–3.7
UTI and ASB	57	5 (9)	1.5	0.59–3.9
UTI and recurrent UTI	19	1 (5.3)	0.35	0.00–420

## Data Availability

The data presented in this study are available on request from the corresponding author. The data are not publicly available due to privacy reasons.
